# Species Comparison of Pre-systemic Bioactivation of Vicagrel, a New Acetate Derivative of Clopidogrel

**DOI:** 10.3389/fphar.2016.00366

**Published:** 2016-10-07

**Authors:** Zhi-xia Qiu, Wen-chao Gao, Yu Dai, Su-feng Zhou, Jie Zhao, Yang Lu, Xi-jing Chen, Ning Li

**Affiliations:** ^1^Department of Pharmacology of Chinese Materia Medica, China Pharmaceutical UniversityNanjing, China; ^2^Clinical Pharmacokinetics Research Laboratory, School of Basic Medicine and Clinical Pharmacy, China Pharmaceutical UniversityNanjing, China; ^3^National Experimental Teaching Demonstration Center of Pharmacy, China Pharmaceutical UniversityNanjing, China

**Keywords:** vicagrel, active metabolite, inactivation hydrolysis, esterase, CYP450s, bioactivation, species difference

## Abstract

Previously we have found vicagrel, a new acetate derivative of clopidogrel, underwent hydrolysis to 2-oxo-clopidogrel and subsequent conversions to its pharmacological active metabolite (AM) and inactive carboxylic acid metabolite (CAM). This study demonstrated the interspecies differences of the vicagrel bioactivation by comparing the critical vicagrel metabolites formation in rats, dogs and human. The pharmacokinetic studies with rats and dogs were conducted after intragastric administration of vicagrel, followed by *in vitro* metabolism investigation in venous system, intestinal/hepatic microsomes from rats, dogs and human. An obvious disparity was observed in system exposure to AM (99.0 vs. 635.1 μg⋅h/L, *p* < 0.05) and CAM (10119 vs. 2634 μg⋅h/L, *p* < 0.05) in rats and dogs. It was shown that the cleavage of vicagrel was almost completed in intestine with great different clearance (53.28 vs. 3.643 L⋅h^-1^⋅kg^-1^, *p* < 0.05) in rats and dogs. With no further hydrolysis to CAM, the greatest clearance of AM (3.26 mL⋅h^-1^⋅kg^-1^) was found in dog intestine. In rat plasma, 2-oxo-clopidogrel was much more extensively hydrolyzed to CAM than in dog and human. Albeit similar hydrolysis clearance and AM production was observed among hepatic microsomes of the three species, the production velocity of CAM ranked highest in dogs (7.55 pmol/min/mg protein). Therefore, the unconformity of AM and CAM exposure cross species mainly came from the metabolism of 2-oxo-clopidogrel associated largely with tissue specificity and interspecies differences of esterases. In human, the pharmacokinetics of vicagrel might be more optimistic due to less inactivation hydrolysis before reaching liver.

## Introduction

**FIGURE 1:**
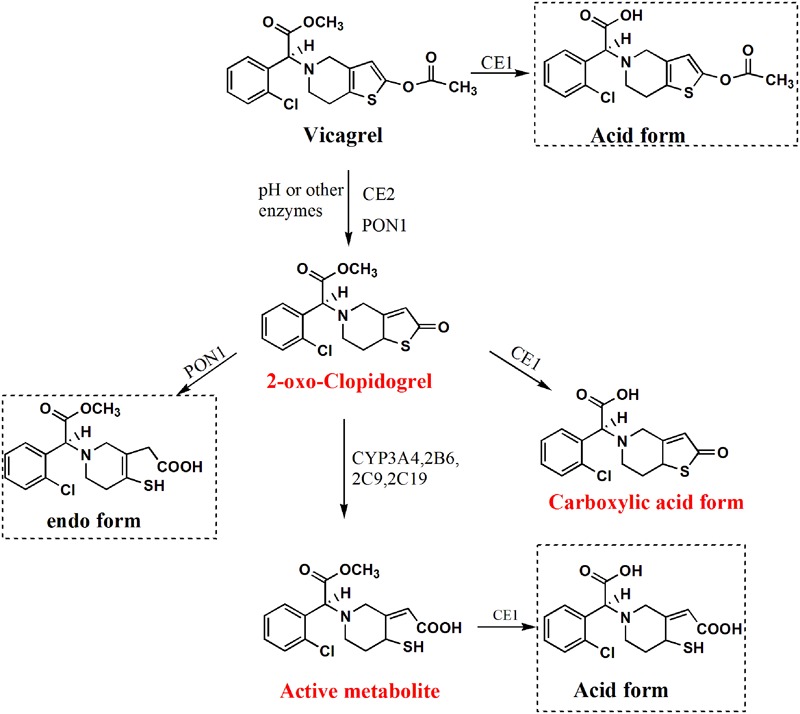
**Chemical structures and proposed metabolic pathway of vicagrel (Representative metabolites surrounded by dashed lines were not considered in the present study; there were three competitive pathways involving 2-oxo-clopidogrel further metabolism, we mainly focused on the critical pathway to generate AM) (Qiu et al., 2014)**.

Currently, the low-response or non-response to clopidogrel treatment in cardiovascular diseases is of increasing concern, particularly in the condition of acute coronary syndrome (ACS) or clopidogrel resistance (CR) (Nguyen et al., 2005; Vlachojannis et al., 2011; Qureshi and Hobson, 2013). Clopidogrel is regularly prescribed in combination with aspirin to treat ACS or prevent thrombotic events following percutaneous coronary intervention (PCI) (Cooke et al., 2006). Albeit this combined therapy is highly recommended for its efficiency on reducing the mortality, it showed potential gastro-intestinal risk such as duodenal ulcer and gastrointestinal bleeding caused by aspirin in the formulation (Cooke et al., 2006). Prasugrel, a modified analog of clopidogrel shows higher pharmacological potency due to its rapid conversion to active metabolite (AM), which allows it to be used as monotherapy for the treatment of ACS (Lazar and Lincoff, 2009; Wiviott et al., 2010). However, US FDA issued a black box warning regarding prasugrel and its side effect of potential bleeding risk accompanied by its fast metabolite activation (FDA, 2010; Rodriguez et al., 2013). Given the merits and demerits of clopidogrel and prasugrel, vicagrel (**Figure 1**), a novel acetate analog of clopidogrel was developed (Shan et al., 2012) and approved by China Food and Drug Administration (CFDA). Vicagrel, sharing the similar metabolic pattern to prasugrel, is esterified from clopidogrel being expected to perform improved anti-platelet efficiency and reduced bleeding risk (Shan et al., 2012).

In preliminary pharmacological screening, vicagrel exhibits enhanced anti-platelet aggregation potency compared to clopidogrel and shows somewhat lower potency than prasugrel (Shan et al., 2012). In addition, the systemic exposure of AM produced from vicagrel was 5–6 fold higher than that from clopidogrel according to our previous study (Qiu et al., 2013). It is inspiring that the AM production efficiency is improved through faster 2-oxo-clopidogrel formation from vicagrel catalyzed by esterases including carboxylesterases (CEs) especially CE2, paraoxonases (PON), butyrylcholineesterases (BChE) (Qiu et al., 2014). This very rapid formation of 2-oxo-clopidogrel from vicagrel facilitates subsequent AM production mediated by CYP450s.

Clearly, CEs are mainly responsible for the hydrolysis of 85% clopidogrel to the main inactive acid form, although there are many other non-esterase hydrolysis enzymes involved in this process (Hagihara et al., 2009; Farid et al., 2010). The inactive hydrolysis may also happen to 2-oxo-clopidogrel (about 50%) and AM (Zhu et al., 2013). With the same main skeleton as clopidogrel, vicagrel could also suffer the inactivation through its hydrolysis to carboxylic acid (**Figure 1**). In order to clarify its biotransformation property, it is vital to look into the species difference of esterases. CE1 is expressed far more abundantly compared to CE2 in the liver of all species including rats, dogs and human (Taketani et al., 2007; Yang et al., 2009). While CE2 but not CE1 is predominately expressed in rat and human intestine (Crow et al., 2007; Taketani et al., 2007). It is well documented that there is almost no any esterase located in the intestine of dogs (Berry et al., 2009). It is worth to point out that rat plasma contains almost all types of esterase such as CEs (CE1 and CE2), PON, BChE, ArE, and AChE (Satoh and Hosokawa, 2006; Bahar et al., 2012). However, there are no CEs existed in the plasma of dogs and human at all (Williams et al., 2011). Based on the remarkable species differences in esterases distribution, which may affect the metabolism and disposition of ester compounds, the present investigation aimed to evaluate the vicagrel disposition in rats and dogs to suggest its possible metabolic fate in human. In the current study, the pharmacokinetic behaviors of 2-oxo-clopidogrel, carboxylic acid metabolite (CAM) of 2-oxo-clopidogrel, and AM in rats and dogs following intragastric administration of vicagrel were demonstrated. Additionally, the *in vitro* hydrolysis mainly focusing on esterases was assessed in plasma, intestinal and hepatic microsomes from rats, dogs and human. Moreover, the CYP450s-dependent oxidation investigation was carried out in intestinal and hepatic microsomes from rats, dogs and human to compare the enzyme affinity and AM production velocity. This study shed light on *in vivo* or *in vitro* pre-systemic pharmacokinetic behaviors of vicagrel in different species; further pre-clinical study is needed to provide a better prediction of its metabolic fate and pharmacological response in human.

## Materials and Methods

### Chemicals and Reagents

Vicagrel (free base, 98% pure), 2-oxo-clopidogrel (99% pure) were kindly supplied by the State Key Laboratory of Natural Medicines and the Center of Drug Discovery, China Pharmaceutical University, respectively. MP-AM (AM derivatized with 3-methoxyphenacyl bromide) and CAM of 2-oxo-clopidogrel was synthesized by the Center of Drug Discovery, China Pharmaceutical University. Prasugrel thiolactone metabolite (R-95913, 98% pure) was donated by Chia-Tai Tian Qing Pharmaceutical Co. Ltd (Jiangsu, China), and used as internal standard (ISTD) in the present experiment. The derivatizing agent 3-methoxyphenacyl bromide (MPBr) was purchased from TCI (Shanghai) Development Co., Ltd. Glutathione (GSH) was bought from J&K Scientific (Shanghai, China). Sodium carboxymethyl cellulose (CMC-Na) was purchased from Dai-Ichi Kogyo Seiyaku Co., Ltd (Shanghai, China). Nicotinamide adenine dinucleotide phosphate (NADPH) regenerating system was bought from Sigma-Aldrich (St. Louis, MO, USA). HPLC grade acetonitrile and methanol were purchased from Tedia (Fairfield, OH, USA). All other chemicals and agents were of analytical grade.

### Rat, Dog, and Human Intestinal and Hepatic Microsomes

The pooled intestinal microsomes of rats [20 male Sprague-Dawley (SD) rats] and dogs (5 male beagle dogs) were kindly supplied by Research Institute for Liver Disease (Shanghai, China). The human intestinal microsomes (pooled, 10 mixed gender donors) were purchased from Celsis In Vitro Technologies (Baltimore, MD, USA). The human hepatic microsomes (pooled, 10 male donors) were supplied by Research Institute for Liver Disease (Shanghai, China). The rat (pooled 10 male and female SD rats) and dog (pooled 10 male and female beagle dogs) hepatic microsomes were prepared within lab by differential centrifugation at 9000 g at 4°C for 20 min and 100,000 *g* at 4°C for 60 min. The protein concentration of produced microsomes was determined by BCA protein assay commercial kit from Shanghai Generay Biotech Co., Ltd (Shanghai, China). The microsomes were kept at -70°C and thawed at 4°C before use.

### Rat, Dog and Human Plasma

Rat plasma was sampled and pooled from 10 male and female SD rats purchased from Shanghai SIPPR/BK Experimental Animal Co., Ltd (Shanghai, China). Dog plasma was collected and pooled from 10 male and female beagle dogs housed in Southeast University Laboratory Animal Center (Nanjing, China). Human plasma (pooled, 10 mixed gender donors) was kindly offered by Nanjing First Hospital (Nanjing, China). The plasma was stored at -70°C till analysis.

### Experimental Animals

Sprague-Dawley rats of both sexes (200 ± 20 g) were supplied by Shanghai SIPPR/BK Experimental Animal Co., Ltd (Shanghai, China). Healthy adult beagle dogs of both sexes were kept in Laboratory Animal Center of Southeast University (Nanjing, China). The rats and dogs were housed under humanized conditions with free access to water and food and acclimated to the living environment (temperature: 20 ± 2°C, relative humidity: 50 ± 20%) for 1 week prior to experiments. Ahead of drug administration, the rats and dogs were fasted for 12 h with free access to water. All the experiments were conducted under the protocol of Animal Ethics Committee of China Pharmaceutical University.

### Pharmacokinetic Study in Rats and Dogs

As previously described (Qiu et al., 2013), vicagrel suspended in CMC-Na solution was intragastrically administrated to rats at 50 μmol/kg and dogs at 19.3 μmol/kg. After administration, about 100 μL of blood was withdrawn from rat retinal venous plexus at 0, 0.25, 0.5, 0.75, 1, 2, 3, 4, 6, 8, 12, and 24 h. For dogs, about 0.5 mL of blood was collected into heparinized tubes via forearm vein at 0, 0.083, 0.25, 0.5, 0.75, 1, 2, 4, 6, 8, 12, and 24 h. The blood samples were immediately centrifuged at 12,000 *g* at 4°C for 1 min. The harvested plasma was processed by the addition of triple volume of cold acetonitrile (containing 50 ng/mL ISTD, 5% acetic acid and 10 mM MPBr) to avoid the possible degradation caused by plasma proteins and derivatize AM (Qiu et al., 2013). Also, 1,4-dithio-DL-threitol (DTT, as antioxidant) significantly increased the stability of 2-oxo-clopidogrel (Li et al., 2015). In our study, the acidification by acetic acid could also prevent the degradation of 2-oxo-clopidogrel (from below 50% to above 85%) by compared to the samples without acetic acid treatment. 2-oxo-clopidogrel (89.3, 93.1, 99.3%) and AM (87.2, 98.1, 102.2%) remained stable up to 12 h at 4°C, and 15 days in -70°C storage with less than 15% deviation from nominal concentrations at QC levels. The processed plasma samples were vortexed for 3 min, placed at room temperature for 10 min, and centrifuged at 16,000 rpm at 4°C for 10 min. The supernatants of prepared samples were stored at -70°C till analysis. An aliquot of 5 μL of the supernatant was injected for LC-MS/MS analysis.

### Pharmacokinetic Parameters of Target Metabolites in Rats and Dogs

A non-compartmental analysis was applied to assess the pharmacokinetic behaviors in rats or dogs. The maximum concentration of target metabolites (*C*_max_) and the time to reach maximum concentration (*T*_max_) were directly obtained from the observed data. The area under the plasma concentration-time curve from 0 to the last time point (AUC_0-t_) and to the infinity (AUC_0-∞_) was calculated via trapezoidal rule. The mean retention time (MRT) was also estimated. The half-life (*t*_1/2_) was estimated via non-parametric elimination rate constant k. The parameters were calculated using WinNonlin (Version 6.4; Pharsight Corp., Mountain View, CA, USA).

In order to investigate and compare the transformation extent of AM and CAM from 2-oxo-clopidogrel in rats and dogs after oral administration of vicagrel, a production efficiency (E) was put forward between the system exposure of AM or CAM and 2-oxo-clopidogrel, a good evidence of pre-systematic formation of AM or CAM from 2-oxo-clopidogrel.

$$E_{\mathrm{AM}}=\frac{{\mathrm{AUC}}_{0-\infty \mathrm{AM}}}{{\mathrm{AUC}}_{0-\infty 2-oxo-clopidogrel}}\;{or E}_{\mathrm{CAM}}=\frac{{\mathrm{AUC}}_{0-\infty \mathrm{CAM}}}{{\mathrm{AUC}}_{0-\infty 2-oxo-clopidogrel}}$$

### *In vitro* Metabolism of Vicagrel and 2-Oxo-clopidogrel in the Intestinal Microsomes

The incubation was conducted in the intestinal microsomes to study the degradation of vicagrel or 2-oxo-clopidogrel in the intestine. The protein concentrations of intestinal microsomes were set at 20, 50, and 50 μg protein/ml for vicagrel hydrolysis in rats, dogs and human, respectively. The reaction mixtures was pre-incubated with proteins in Tris buffer (pH 7.4, 50 mM) at 37°C, and initiated by the addition of vicagrel with a final concentration at 10 μM. The reactions were terminated at 0, 1, 2, 5, 10, 20, 30, 60, 90, and 120 min by adding double volume of cold acetonitrile (containing 50 ng/ml ISTD and 5% acetic acid).

In order to investigate the contribution of CYP450s in the intestine to 2-oxo-clopidogrel cleavage, the incubation mixtures contained intestinal microsomes, MgCl_2_ (5 mM), NADPH (5 mM), GSH (10 mM), and the reaction was initiated at 37°C by adding 2-oxo-clopidogrel with a final concentration at 10 μM. At 0, 5, 10, 20, 30, 60, 90, and 120 min, the reaction was stopped and the samples were derivatized as described above. The intestinal protein concentration was all set at 1 mg/ml for rats, dogs and human. The added GSH was to generate and stabilize AM (2-oxo-clopidogrel is converted to a sulfenic acid intermediate, which is further reduced by GSH to open the thiolactone ring to produce a GSH conjugate, then to yield AM by a thiol-disulfide exchange) (Zhang et al., 2012, 2013). Meanwhile, the contribution of intestinal CYP450s-mediated metabolism was assessed by the reaction with or without the presence of NADPH.

### *In vitro* Hydrolysis of 2-Oxo-clopidogrel in Plasma

Before the *in vitro* hydrolysis assay, plasma was centrifuged at 3000 rpm for 10 min at 4°C, and the supernatant was adjusted to pH 7.4 using Tris buffer (pH 7.4, 0.05 M). For the subsequent hydrolysis of 2-oxo-clopidogrel, the plasma volume was 0.1, 10, and 10% of the total incubation volume for rats, dogs, and human, respectively. The reaction was terminated at 0, 2, 5, 10, 20, 30, 60, 90, and 120 min as described above. Then the sample was processed for analysis.

### *In vitro* Metabolism of 2-Oxo-clopidogrel in the Hepatic Microsomes

Initially, the hydrolytic reaction was carried out with hepatic microsomes to assess the degradation of 2-oxo-clopidogrel without the cofactor NADPH. The protein concentrations of hepatic microsomes were 0.5, 0.5 and 0.2 mg/ml for rats, dogs and human, respectively. The incubation mixtures was pre-incubated in Tris buffer (pH 7.4, 50 mM) at 37°C, and initiated by addition of 2-oxo-clopidogrel (final concentration at 10 μM). The reactions were terminated at 0, 5, 10, 20, 30, 60, 90, and 120 min as described above. For the system of AM production, the mixtures contained protein (1 mg/ml for rats and dogs, 0.5 mg/ml for human), MgCl_2_, NADPH, GSH, and the incubations were initiated by adding of 2-oxo-clopidogrel (final concentration at 10 μM). At 0, 5, 10, 20, 30, 60, 90, and 120 min, the reaction was stopped and derivatized as above method.

### *In vitro-In vivo* Extrapolation

In the *in vitro* hydrolysis or NADPH-mediated oxidation experiments, the incubation time and protein amounts were varied among species and organs. In order to compare the metabolic profiles of vicagrel or 2-oxo-clopidogrel among rats, dogs and human, we introduced the *in vitro* clearance, Cl*_invitro_* by scaled in organ level or the whole body in terms of kinetics, which were estimated by the following equation:

$$C1_{in\;\;vitro}=\frac{0.693}{t_{1/2}}(\min^{-1})\times \frac{V_{\mathrm{incubation}(\mathrm{ml})}}{\mathrm{Pr}{\mathrm{otein}}_{\left(\mathrm{mg}\;\mathrm{protein}\right)}}$$

Where *t*_1/2_ was the half-life estimated from the elimination rate constant λ of vicagrel or 2-oxo-clopidogrel in plasma, intestinal and hepatic microsomes from rats, dogs or human. Protein was the protein concentration of plasma, intestinal or hepatic microsomes in the incubation system. V was the total volume of the incubation system. Afterward, the Cl*_invitro_* was scaled to intrinsic clearance (Cl_int_) by introducing the scaling factors, calculated as follows:

$$C1_{int}=C1_{in\;\;vitro}\times \frac{\mathrm{Pr}{\mathrm{otein}}_{\left(\mathrm{mg}\;\mathrm{protein}\right)}}{{\mathrm{Tissue}}_{\left(g\right)}}\times \frac{{\mathrm{Tissue}}_{\left(g\right)}}{{Body weight}_{\left(\mathrm{kg}\right)}}$$

Tissue represented the weight of intestine or liver, or plasma volume, where the intestine weight was set at 4, 220, 700 g (Karlsson et al., 2013), and liver weight was 10, 320, and 1800 g for rats, dogs and human respectively (Davies and Morris, 1993). The volume of plasma in rats, dogs and human was referred as 7.8, 515, 3000 ml, respectively. The microsomal protein yield ratio was 3 mg/g for intestine and 45 mg/g for liver (Obach et al., 1997; Paine et al., 1997; Yang et al., 2007). The plasma total protein was set at 65, 58, and 78 mg protein per plasma unit for rats, dogs and human, respectively (Tibbitts, 2003). The other parameters referred to physiological parameters in lab in laboratory rats, dogs and human.

### AM Formation Kinetics Assay in the Intestinal and Hepatic Microsomes

For AM production, the triplicate reaction mixture consisted of Tris buffer (pH 7.4, 50 mM), 5 mM NADPH, 5 mM MgCl_2_, 5 mM GSH, human, dog, or rat intestinal or hepatic microsomes. The substrate concentration of 2-oxo-clopidogrel was set at 0.5, 1, 2, 5, 10, 20, 50, and 100 μM. The final protein concentrations of microsomes were optimized as 1.0 and 0.5 mg protein/ml, and the incubation time was set at 60 min and 30 min for intestinal and hepatic microsomes, respectively. After the incubation, the reaction was terminated by the addition of cold acetonitrile containing ISTD and derivatization reagent.

The production pattern in the intestinal and hepatic microsomes was fitted to Michaelis–Menten equation shown as below. Meanwhile, the profile of productive velocity versus substrate was transformed via Eadie–Hofstee plot.

$$v=\frac{v_{\max}\times S}{K_{m}+S}\;(Michaelis-Menten\;\mathrm{equation})$$

Where *v* was the formation velocity of AM, expressed as pmol/min/mg protein, likewise, V_max_ was the maximum formation velocity, S presented the substrate concentration, K_m_ was the Michaelis constant, represented the substrate concentration when the reaction velocity reaching the half of V_max_, also, K represented the enzyme affinity to substrate. The Eadie-Hofstee plot was used to evaluate the authentic AM formation kinetics whether the formation exhibited monophasic or not. The data was handled by Graphpad Prism 5.0. The *in vitro* clearance and intrinsic clearance of 2-oxo-clopidogrel to AM were introduced to evaluate the contributions of CYP-based oxidation to AM from 2-oxo-clopidogrel, where Cl_in vitro_ was calculated as Cl_in vitro_ = 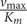, and the intrinsic clearance was scaled using *in vitro t*_*1/2*_ approach.

### LC-MS/MS Analysis

Vicagrel, 2-oxo-clopidogrel, CAM, AM and ISTD were analyzed via LC-MS/MS analysis according to our previous report (Qiu et al., 2013). Specifically, the analysis used an API4000 MS system and Shimadzu HPLC system. The analytes were separated via gradient elution on Shimadzu VP-ODS column, and then flowed with mobile phase to MS system to conduct MRM (multiple reaction monitoring) scan. The column temperature was set at 30°C. The transition conditions of specific analyte were optimized. Vicagrel, 2-oxo-clopidogrel, CAM, AM-MPBr (AM was derivatized by MPBr), as well as ISTD were monitored as *m*/*z* 380.1 to *m*/*z* 212.1, *m*/*z* 338.2 to *m*/*z* 155.1, *m*/*z* 324 *to m*/*z* 169.2, *m*/*z* 504.0 to *m*/*z* 212.1, and 332.1 to *m*/*z* 149.1, respectively. The declustering potential was set at 45.0, 45.0, 53.1, 36.5, and 45.0 v, respectively, and the collision energy at 35, 35, 38.1, 25.4 and 35 ev, respectively. Each run was 7.5 min. Meanwhile, the quality control samples were analyzed to ensure the accuracy and precision of analytical method.

### Statistical Analysis

All data were represented as the means ± SEM. Statistical analysis was calculated by a one-way ANOVA with Newman–Keuls test. The acceptable level of significance was established at *p* < 0.05.

## Results

### Pharmacokinetics of Target Metabolites of Vicagrel in Rats and Dogs

**FIGURE 2:**
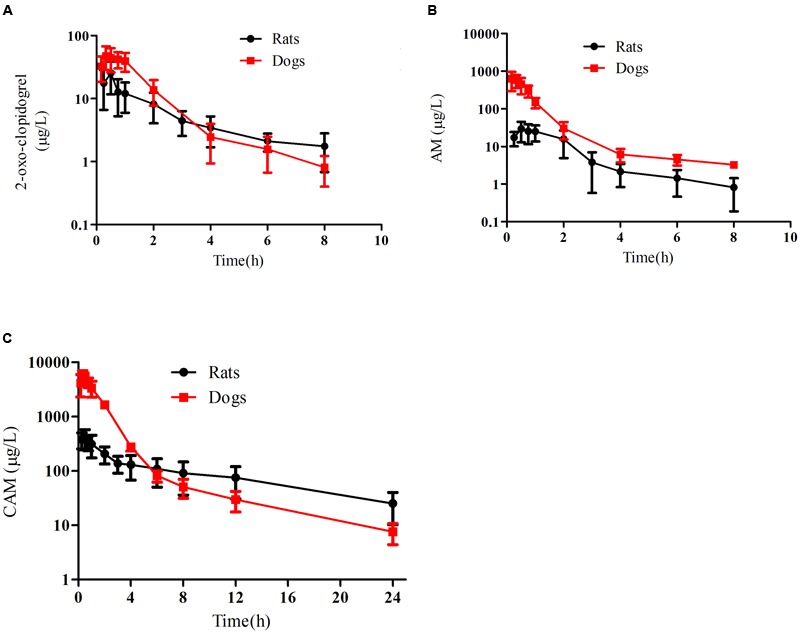
**Plasma concentration-time profiles of 2-oxo-clopidogrel **(A)**, AM **(B)** and carboxylic acid metabolite **(C)** in dogs (19.4 μmol/kg, *n* = 5) and rats (50 μmol/kg, *n* = 5) following intragastric administration of vicagrel.** (The line marked in red represented the profiles in dog).

Following intragastric administration to rats or dogs, vicagrel was rapidly transformed to 2-oxo-clopidogrel, AM and CAM (**Figure 2**). In rats and dogs, 2-oxo-clopidogrel reached each *C*_max_ within 0.45 ± 0.23 and 0.61 ± 0.32 h, respectively, and the AUC_0-∞_ of 2-oxo-clopidogrel in dogs was about two-fold higher than that in rats. AM exhibited shorter peak time (0.16 ± 0.09 h) and higher *C*_max_ (713.1 ± 332.5 μg/L) in dogs, compared to those in rats (0.67 ± 0.24 h, 39.7 ± 23.2 μg/L). Correspondingly, the system exposure to AM was much higher in dogs (716.5 ± 238.5 μg⋅h/L) than that in rats (64.2 ± 24.2 μg⋅h/L). The E_AM_ (production efficiency) was about 1.2 in rats and 6.5 in dogs. The CAM in rat peaks at 0.50 ± 0.27 h with *C*_max_ at 372.9 ± 96.84 μg/L, while that in dog peaks at 0.35 ± 0.14 h with *C*_max_ at 5676.6 ± 388.7 μg/L. The AUC_0-∞_ of CAM was 3080 ± 641.9 μg⋅h/L with an estimated half-life of 8.86 ± 1.23 h in rats. Surprisingly, AUC_0-∞_ of CAM in dogs were much higher, calculated at 10163 ± 1179.4 μg⋅h/L. Additionally, it was cleaved more rapidly with a half-life of 4.97 ± 0.97 h. And the E_CAM_ was calculated as 59.5 in rats and 92.2 in dogs (**Table 1**).

**Table 1:** Pharmacokinetic parameters of 2-oxo-clopidogrel, AM and CAM following intragastric administration of vicagrel at 50 μmol/kg to rats (*n* = 6) and 19.4 μmol/kg to dogs (*n* = 6).

	2-Oxo-clopidogrel		AM		CAM	
	Rats	Dogs	Rats	Dogs	Rats	Dogs
C_max_(μg/L)	22.6 ± 9.79	64.8 ± 8.04^∗^	39.7 ± 23.2	713.1 ± 332.5^∗^	372.9 ± 96.84	5676.6 ± 388.7^∗^
T_max_(h)	0.45 ± 0.23	0.61 ± 0.32	0.67 ± 0.24	0.16 ± 0.09 ^∗^	0.50 ± 0.27	0.35 ± 0.14
AUC_0-t_(μg⋅h/L)	47.9 ± 15.8	109.8 ± 50.3^∗^	62.0 ± 23.8	711.9 ± 239.1^∗^	2634 ± 568.9	10119 ± 1193.6^∗^
AUC_0-∞_(μg⋅h/L)	51.8 ± 17.1	110.2 ± 50.4^∗^	64.2 ± 24.2	716.5 ± 238.5^∗^	3080 ± 641.9	10163 ± 1179.4^∗^
MRT_0-t_(h)	3.45 ± 0.46	1.33 ± 0.45^∗^	1.96 ± 0.45	0.97 ± 0.09^∗^	7.47 ± 1.21	1.69 ± 0.27^∗^
*t*_1/2_(h)	3.32 ± 0.93	0.84 ± 0.42^∗^	1.85 ± 1.17	1.19 ± 0.28^∗^	8.86 ± 1.23	4.97 ± 0.97^∗^

*^∗^*p* < 0.05, rat vs. dog.*

### Hydrolysis of Vicagrel and 2-Oxo-clopidogrel in the Intestinal Microsomes

**FIGURE 3:**
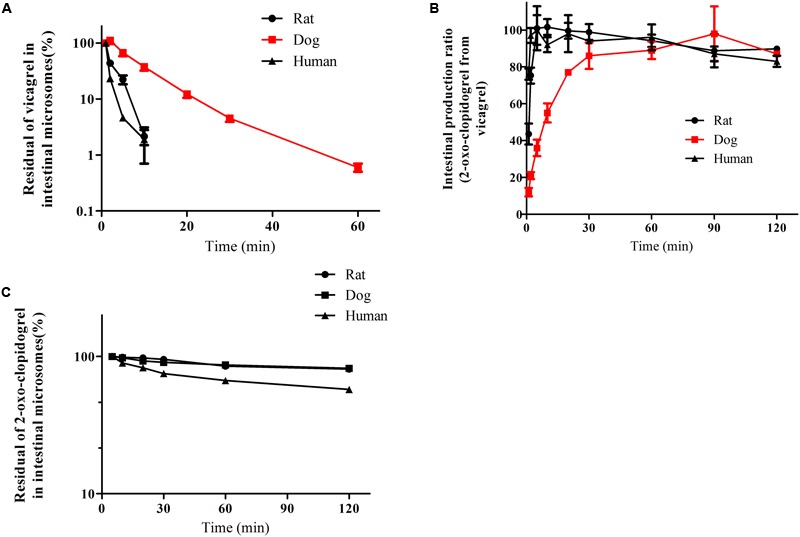
**The degradation profiles of vicagrel **(A)**, 2-oxo-clopidogrel **(B)** and production profiles of 2-oxo-clopidogrel from vicagrel **(C)** in the intestinal microsomes from rat, dog and human (The reactions were conducted in pH 7.4 Tris buffer at 37°C and initiated by the addition of vicagrel with a final concentration at 10 μM; The line marked in red represented the profiles in dog)**.

After the initiation of hydrolysis, vicagrel was rapidly hydrolyzed in the intestinal fractions (**Figure 3A**). In rat and human intestinal microsomes, the 2-oxo-clopidogrel was produced immediately from vicagrel and peaked around at 5–10 min with almost complete conversion (the observed response under initial condition without incubation was treated as control). While in dog intestinal microsomes, the amount of 2-oxo-clopidogrel was gradually increased upon the incubation time with complete cleavage from vicagrel at approximate 30 min (**Figure 3B**). The degradation half-life of vicagrel in the intestinal microsomes from rats, dogs and human were estimated as 2.4 ± 0.2, 15.3 ± 2.6 and 1.6 ± 0.7 min, respectively. The scaled Cl_int_ was highest in rats (53.28 L⋅h^-1^⋅kg^-1^), and lowest in dogs (3.643 L⋅h^-1^⋅kg^-1^). It is evidenced that the spiked 2-oxo-clopidogrel in the same incubation systems was relatively stable in the absence of NADPH (**Figure 3C**, **Table 2**), which was in exceptional agreement with the below results that there was no CAM generated in the intestinal microsomes from rats, dogs or human, probably due to the lack of CE1 expression in the intestine of the three species.

**Table 2:** Intestinal hydrolysis of vicagrel and 2-oxo-clopidogrel in the intestinal microsomes from rats, dogs, and human.

	λ (min^-1^)	*t*_1/2_ (min^-1^)	Cl*_invitro_* (L⋅h^-1^⋅g protein^-1^)	Cl_int_ (L⋅h^-1^⋅kg^-1^)
**Vicagrel**				
Rat	0.296 ± 0.020	2.4 ± 0.2	888.0 ± 60.00	53.28 ± 3.600
Dog	0.046 ± 0.007^∗^	15.3 ± 2.6^∗^	55.20 ± 8.651^∗^	3.643 ± 0.571^∗^
Human	0.439 ± 0.020	1.6 ± 0.7	526.8 ± 24.00	15.80 ± 0.720
**2-oxo-clopidogrel**				
Rat	0.0023 ± 0.000	–	–	–
Dog	0.0030 ± 0.001	–	–	–
Human	0.0018 ± 0.003	–	–	–

*–The parameters were not calculated, since the elimination rate constants of 2-oxo-clopidogrel in the intestinal microsomes without NADPH were restricted within quite small extent. ^∗^*p* < 0.05, dog vs. rat or human.*

### Hydrolysis of 2-Oxo-clopidogrel in Plasma

In plasma, 2-oxo-clopidogrel was degraded with the elimination half-life of 66.4 ± 14.0, 134.6 ± 16.9 and 127.1 ± 16.3 min in rats, dogs and human, respectively (**Table 3**). Correspondingly, the scaled Clint ranked the highest in rat plasma (0.182 L⋅h^-1^⋅kg^-1^), followed by dog (0.075 L⋅h^-1^⋅kg^-1^) and human (0.053 L⋅h^-1^⋅kg^-1^).

**Table 3:** Plasma hydrolysis of 2-oxo-clopidogrel in rats, dogs, and human.

	λ (min^-1^)	*t*_1/2_ (min^-1^)	Cl*_invitro_* (L⋅h^-1^⋅g protein^-1^)	Cl_int_ (L⋅h^-1^⋅kg^-1^)
Rat	0.011 ± 0.0019	66.4 ± 14.0^∗^	0.101 ± 0.019^∗^	0.182 ± 0.034^∗^
Dog	0.0052 ± 0.0012	134.6 ± 16.9	0.052 ± 0.010	0.075 ± 0.014
Human	0.0063 ± 0.0011	127.1 ± 16.3	0.046 ± 0.008	0.053 ± 0.009

*^∗^*p* < 0.05, rat vs. dog or human.*

Besides, only in rat plasma CAM was extensively produced, and the formation was dose-dependent with 11.05 and 34.65 pmol/min/mg protein at 5 and 50 μM (**Table 5**).

### Hydrolysis of 2-Oxo-clopidogrel in Hepatic Microsomes

The degradation half-life of 2-oxo-clopidogrel in rat, dog, and human hepatic microsomes was 1.09 ± 0.06, 1.34 ± 0.09 and 2.88 ± 0.42 h^-1^, respectively, and the scaled intrinsic Cl_int_ was 2.304 ± 0.125, 1.498 ± 0.100 and 1.412 ± 0.209 L/h/kg, respectively (**Table 4**).

**Table 4:** Hepatic Hydrolysis of 2-oxo-clopidogrel in hepatic microsomes from rats, dogs and human.

	λ (h^-1^)	*t*_1/2_ (h^-1^)	Cl*_invitro_* (L⋅h^-1^⋅g protein^-1^)	Cl_int_ (L⋅h^-1^⋅kg^-1^)
Rat	0.640 ± 0.035	1.09 ± 0.06^∗^	1.280 ± 0.069	2.304 ± 0.125
Dog	0.520 ± 0.035	1.34 ± 0.09	1.036 ± 0.066	1.498 ± 0.100
Human	0.244 ± 0.036	2.88 ± 0.42	1.220 ± 0.181	1.412 ± 0.209

*^∗^*p* < 0.05, rat vs. dog or human.*

Carboxylic acid metabolite was extensively generated from 2-oxo-clopidogrel in hepatic microsomes in the absence of NADPH. The dog hepatic microsomes exhibited the greatest production rate, while rats ranked the lowest among the three species. At 5 μM of 2-oxo-clopidogrel, the CAM production rates were calculated at around 1.78, 7.55 and 4.14 pmol/min/mg protein for rats, dogs, and human, respectively (**Table 5**).

**Table 5:** The production velocity of CAM from 2-oxo-clopidogrel in the plasma, intestinal and hepatic microsomes from rats, dogs, and human.

		Rats	Dogs	Human
**Production velocity (pmol/min/mg protein)**				
Intestine	5 μM	–	–	–
	50 μM	–	–	–
Plasma	5 μM	11.05 ± 0.55	–	–
	50 μM	34.65 ± 0.68	–	–
Liver	5 μM	1.78 ± 0.63	7.55 ± 0.68^∗^	4.14 ± 0.78
	50 μM	35.13 ± 3.34	67.13 ± 7.56^∗^	43.88 ± 2.56

*–The parameters were not calculated, because there was no CAM produced in the plasma of dogs or human, or the intestinal microsomes of rats, dogs and human. ^∗^*p* < 0.05, rat vs. dog.*

### AM Formation Kinetics in the Intestinal and Hepatic Microsomes

**FIGURE 4:**
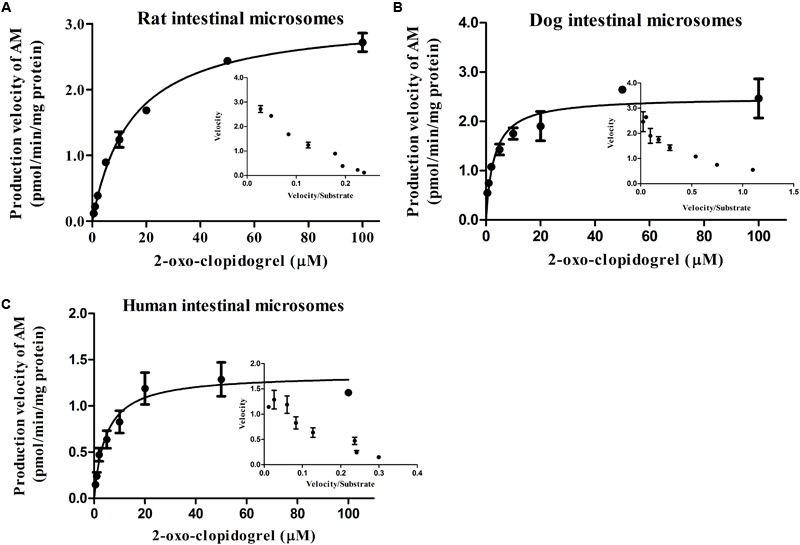
**Formation kinetics of AM in the intestinal microsomes from rat **(A)**, dog **(B)**, and human **(C)**.** The inset figures depicted the Eadie–Hofstee plots of AM (The reactions were performed in pH 7.4 Tris buffer at 37°C; the incubation time was set at 60 min).

**FIGURE 5:**
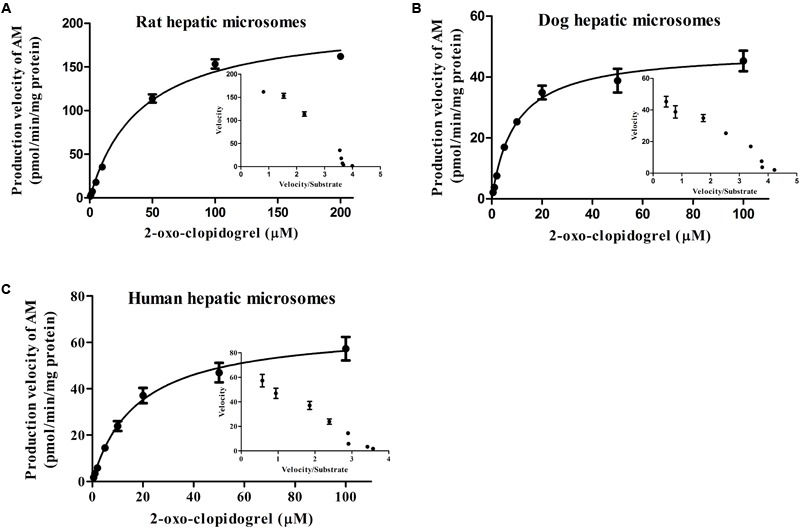
**Formation kinetics of AM in the hepatic microsomes from rat **(A)**, dog **(B)**, and human **(C)**.** The inset figures depicted the Eadie–Hofstee plots of AM (The reactions were performed in pH 7.4 Tris buffer at 37°C; the incubation time was set at 30 min).

The well fitted Eadie–Hofstee plots indicated the classic Michaelis-Menten pattern of AM production from 2-oxo-clopidogrel in the presence of NADPH in the intestinal or hepatic microsomes (**Figures 4** and **5**). AM was produced with the Michaelis–Menten rate constant (K_m_) of 14.57, 3.023 and 4.661 μM in intestinal microsomes, and 40.71, 9.392 and 17.58 μM in hepatic microsomes of rats, dogs and human, respectively. The dog’s intestine exhibited the highest intrinsic clearance from 2-oxo-clopidogrel to AM, estimated as 3.26 ml⋅h^-1^⋅kg^-1^. However, there was no significant difference in the hepatic clearance among rats, dogs, and human (**Table 6**).

**Table 6:** Kinetics of AM production from 2-oxo-clopidogrel in the intestinal and hepatic microsomes from rats, dogs, and human.

Intestine	K_m_ (μM)	V_max_ (pmol/min/mg protein)	Cl*_invitro_* (ml⋅h^-1^⋅g protein^-1^)	Cl_int_ (ml⋅h^-1^⋅kg^-1^)
Rat	14.57 ± 1.242	3.102 ± 0.08735	12.77 ± 1.039	0.766 ± 0.0623
Dog	3.023 ± 0.3466	2.489 ± 0.1154	49.40 ± 15.68^∗^	3.26 ± 1.034^∗^
Human	4.661 ± 1.436	1.344 ± 0.1179	17.30 ± 4.414	0.571 ± 0.132

**Liver**	**K_m_ (μM)**	**V_max_ (pmol/min/mg protein)**	**Cl*_invitro_* (L⋅h^-1^⋅g protein^-1^)**	**Cl_int_ (L⋅h^-1^⋅kg^-1^)**

Rat	40.71 ± 4.510	203.4 ± 7.241	0.270 ± 0.0136	0.486 ± 0.025
Dog	9.392 ± 1.194	48.68 ± 1.842	0.315 ± 0.0399	0.453 ± 0.058
Human	17.58 ± 3.294	66.29 ± 2.494	0.233 ± 0.0489	0.269 ± 0.058

*^∗^*p* < 0.05, dog vs. rat or human.*

## Discussion

For clopidogrel, the CYP450-mediated activation to AM is a quantitatively minor metabolic pathway. Parallelly, the majority of clopidogrel is hydrolyzed to clopidogrel CAM, which is subsequently conjugated to clopidogrel acyl glucuronide mediated by β-glucuronidase (Savu et al., 2016). Vicagrel experiences several metabolic fates *in vivo*. It bypasses the first-step of thiolactone metabolite production mediated by CYP1A2, 2C9, 2C19, and 3A4 (Kazui et al., 2010) that are responsible for 2-oxo-clopidogrel production from clopidogrel. Instead, it is converted to 2-oxo-clopidogrel mainly by esterases such as CEs, PON and BChE, guaranteeing the enhanced rate and capacity of 2-oxo-clopidogrel and subsequent AM formation (Qiu et al., 2014). The 2-oxo-clopidogrel is further cleaved to CAM mainly mediated by CE1 (Zhu et al., 2013), which is a parallel metabolic pathway to the AM production mediated by CYP3A4, 2B6, 2C19, and 2C9 (Zhu and Zhou, 2013). Similarly, both vicagrel and AM are also possibly hydrolyzed to their acid forms (Dansette et al., 2012), which presumed to be neglected in the present study (**Figure 1**). Moreover, the generation of AM is an important and direct indicator to distinguish vicagrel and clopidogrel. Hence, 2-oxo-clopidogrel (the most important intermediate metabolite of vicagrel), CAM (the main inactive metabolite of vicagrel) and AM (pharmacological AM) were used as the target metabolites in this article to investigate the disposition of vicagrel in animal species and to suggest its possible metabolic fate in human.

The *in vivo* data showed that the system exposure and transformation efficiency of CAM and AM were both higher in dogs than in rats. However, the duration of CAM retained in rats was dramatically longer than in dogs (MRT 7.47 vs. 1.69 h). Moreover, the AM in dogs was eliminated with a much shorter half-life (Qiu et al., 2013). Thus, the production rather than elimination was the determinant of vicagrel transformation efficiency to AM or CAM in dogs and rats. The further transformation investigation was conducted with comprehensive *in vitro* methods, including the intestinal, hepatic and even pre-hepatic venous system in rats, dogs and human, to clarify the difference of target metabolites production efficiency.

In the cellular absorption assay, vicagrel was found to be linearly absorbed in a time- and dose-dependent manner with passive diffusion as the major absorption pattern (within lab data). Our results indicated that the absorption of vicagrel had little impact on the production rates of AM and CAM. Instead, the intestinal and hepatic metabolism of vicagrel might play a vital role in the differential biotransformation among species.

In spite of the almost quantitatively conversions in all three species, vicagrel was immediately hydrolyzed to its thiolactone metabolite in rat and human intestinal microsomes, rather than in a progressive manner as in dogs. The difference in conversion rates was mainly attributed to the localization and expression pattern of esterases or esterase-like enzymes in the intestine (Qiu et al., 2014). Our previous findings showed that the intestinal CE2 in rats and human responsible for the rapid production of 2-oxo-clopidogrel, exhibited high competence in vicagrel hydrolysis compared to CE1 (Satoh and Hosokawa, 2006; Qiu et al., 2014). While for dogs, there are no esterases identified in the intestine (Berry et al., 2009). Hence, there might be other unidentified proteins in canine intestine involved in the process of vicagrel hydrolysis that works slowly but almost completely. Encouragingly, the clearance pattern of vicagrel to 2-oxo-clopidogrel was in accordance with the production of thiolactone intermediate (R-95913) from prasugrel in the intestine (Hagihara et al., 2010, 2011). Thus, the impact of intestinal esterases differences between species on vicagrel hydrolysis to 2-oxo-clopidogrel is not of much significance.

It is widely recognized that clopidogrel is converted to 2-oxo-clopidogrel, AM, CAM and other metabolites prior to reaching the systemic circulation. In the intestine of rats, dogs and human, there is no convincing evidence to support the existence of CE1 involved in the hydrolysis of 2-oxo-clopidogrel to inactive CAM. This may provide an explanation for stable 2-oxo-clopidogrel concentrations during the incubation with intestinal microsomes in the absence of NADPH. After the addition of NADPH, 2-oxo-clopidogrel was converted to AM with high affinity but low capacity, which fit well to the classical Michaelis–Menten kinetic model, suggesting the critical involvement of CYP450s in the intestinal clearance of 2-oxo-clopidogrel. Compared to rats, the intestine microsomal enzymes in dogs and human exhibited similar or even higher affinity to this conversion. The intrinsic clearance indicated that AM formation from vicagrel in the human showed no significant difference from that in the rats, but it was significantly less than that in dog (*p* < 0.05). These results indicated that the AM production profile in the human intestine might resemble that in rat when following oral administration. This indication was consistent with the previous finding that human and rat shares similar location and abundance of esterases (CE2) and CYP450s (mainly CYP3A) (Martignoni et al., 2006; Taketani et al., 2007).

Once arriving at mesenteric vein, the metabolism of 2-oxo-clopidogrel and AM took place in the blood pool prior to the hepatic portal vein. It’s worth mentioning that there was no vicagrel found in the rat portal vein or in dog circulation system, further showing the complete conversion of vicagrel prior to its arrival to the liver in rats or the circulation in dogs (Qiu et al., 2013, 2014), so the metabolism of vicagrel missing from the intestine was also negligible. Therefore, species differences in the types and levels of esterases in plasma resulted in different patterns of the 2-oxo-clopidogrel hydrolysis. Rat plasma contains the most ubiquitously located esterases, including CEs (CE1 & CE2), PON (mainly PON1), BChE, AChE, ArE, etc. And the plasma of human and dog share similar profile of these enzymes, including PON, BChE, AChE but no CEs (Bahar et al., 2012). The CE1 in rat plasma was responsible for the further hydrolysis of 2-oxo-clopidogrel and AM to their carboxylic acid forms (Zhu et al., 2013). This result was in consistence with the subsequent observations that CE1 in rat plasma could play a pivotal role in the formation of CAM from 2-oxo-clopidogrel and hydrolytic loss of produced AM during absorption process (no quantitative data), while the hydrolysis was limited in dogs or human plasma due to the absence of CE expression. Consequently, the presence of all 2-oxo-clopidogrel, AM, and CAM was found in rat plasma, while dog and human plasma contained only 2-oxo-clopidogrel and AM but no CAM before reaching the liver, which may indicate that the portion of 2-oxo-clopidogrel arriving at dog and human liver overweighed that of rats. Moreover, it is worth noting that 2-oxo-clopidogrel was oxidized to AM via intestinal CYP450s with higher substrate affinity in the dog intestine, indicating 2-oxo-clopidogrel were converted to AM more completely in dog intestine and experienced no further hydrolysis in dog plasma, which could be a plausible explanation for the higher system exposure and transformation efficiency of AM in dogs observed *in vivo*.

As the most important metabolic organ, liver contains the most abundant esterases (CEs mainly CE1, PON, BChE, AchE), CYP450s and other related enzymes with high activities (Martignoni et al., 2006) (18). When reaching to the liver, 2-oxo-clopidogrel and AM must be extensively metabolized. Whereby 2-oxo-clopidogrel experienced extensive hydrolysis to CAM and oxidation to AM. The formation velocity of CAM varied among the three species with the largest velocity was found in dog hepatic microsomes. For the AM production, although the substrate-enzyme affinity in dogs was comparable to that in human and much higher than that in rats, there was no obvious difference in the clearance of 2-oxo-clopidogrel to AM among rats, dogs, and human. Furthermore, hepatic metabolism profiles of 2-oxo-clopidogrel and AM in human and dogs resembled each other as characterized by similar total clearance of 2-oxo-clopidogrel regardless of the presence of NADPH (**Tables 4** and **6**).

Although the produced 2-oxo-clopidogrel and AM encountered further hydrolysis to their carboxylic acid forms, there was no significant difference in 2-oxo-clopidogrel cleavage to AM between rats and dogs in the liver. In the rats, the loss of 2-oxo-clopidogrel in the plasma further led to the reduced amount of 2-oxo-clopidogrel in the liver, which eventually resulted in the low production of AM and CAM in rats compared to dogs. While in the dogs, the higher AM exposure was associated with the higher intestinal production followed by lower loss in the plasma, and the greater CAM production was related to lower hydrolysis loss of 2-oxo-clopidogrel in the blood prior to the hepatic portal vein and the higher production in the liver. Moreover, the acid forms of vicagrel, 2-oxo-clopidogrel and AM could probably undergo glucuronidation as occurred in clopidogrel metabolism (Savu et al., 2016), which would be performed in following study.

## Conclusion

In summary, our present study focused on the pre-systemic bioactivation of vicagrel in the intestinal, hepatic as well as pre-hepatic venous system in species *in vivo* and *in vitro*. After PO administration to rats and dogs, vicagrel rapidly and completely converted to 2-oxo-clopidogrel in the intestine, and subsequently oxidized to AM via intestinal and hepatic CYP450s. In rats, the produced 2-oxo-clopidogrel and AM in the intestine experienced hydrolysis to inactive acid metabolites in pre-hepatic venous system, which did not take place in dogs, and eventually led to the differences in the exposure of AM and CAM between species. It was reasonable to extrapolate that in human, vicagrel might share a similar metabolic fate in the intestine with that in rats, followed by analogous metabolic deposition of that in dog plasma and liver. Given the metabolic pattern of the thiolactone metabolite in dogs, vicagrel was expected to have the potential to show a more ideal pharmacokinetic behavior with higher systemic exposure of AM and a better pharmacological properties with faster onset of anti-platelet aggregation in human.

## Author Contributions

Z-xQ, X-jC, and NL designed the research. Z-xQ, W-cG, and YD carried out the animal experiment, *in vitro* metabolism assay and data analysis. S-fZ and JZ performed the MS/MS analysis. Z-xQ wrote the manuscript and prepared all these data. YL, X-jC, and NL reviewed the final manuscript. X-jC was the project manager.

## Conflict of Interest Statement

The authors declare that the research was conducted in the absence of any commercial or financial relationships that could be construed as a potential conflict of interest.
